# Correction to Identifying Subgroups of People Living With HIV in British Columbia, Canada Based on Sexually Transmitted and Blood‐Borne Infection Testing and Diagnostic Laboratory Data: A Retrospective Cohort Study

**DOI:** 10.1002/hsr2.73065

**Published:** 2026-08-19

Yonkman A, Emerson SD, Sereda P, Bacani N, Toy J, Lima VD, Montaner JSG. Identifying Subgroups of People Living With HIV in British Columbia, Canada Based on Sexually Transmitted and Blood‐Borne Infection Testing and Diagnostic Laboratory Data: A Retrospective Cohort Study. *Health Science Reports*. 2026; 9:e71801.

The reference “von Schreeb S, Pedersen SK, Christensen H, et al. Questioning risk compensation: pre‐exposure prophylaxis (PrEP) and sexually transmitted infections among men who have sex with men, capital region of Denmark, 2019 to 2022. *Euro Surveill*. 2024;29(13):2300451.” was incorrect and should be replaced with the following reference:

Chow EPF, Grulich AE, Fairley CK. Epidemiology and prevention of sexually transmitted infections in men who have sex with men at risk of HIV. *Lancet HIV*. 2019;6(6):e396–e405.

The corresponding in‐text citation should be updated accordingly.

The following sentence and its corresponding citations were included incorrectly and have been removed:

“For example, studies have shown that coinfection with syphilis is associated with higher odds of virologic failure in ART‐naïve PLWH, as well as lower CD4 count regardless of HIV treatment status [16,17].”

The online version of this article has been corrected accordingly.

We apologize for these errors.

